# A Novel Prognostic Signature Based on Metabolism-Related Genes to Predict Survival and Guide Personalized Treatment for Head and Neck Squamous Carcinoma

**DOI:** 10.3389/fonc.2021.685026

**Published:** 2021-06-14

**Authors:** Ying Li, Youliang Weng, Yuhui Pan, Zongwei Huang, Xiaochuan Chen, Wenquan Hong, Ting Lin, Lihua Wang, Wei Liu, Sufang Qiu

**Affiliations:** Department of Radiation Oncology, Fujian Medical University Cancer Hospital, Fujian Cancer Hospital, Fuzhou, China

**Keywords:** metabolism, prognostic signature, head and neck squamous carcinoma, survival, therapeutic response prediction

## Abstract

Metabolic reprogramming contributes to patient prognosis. Here, we aimed to reveal the comprehensive landscape in metabolism of head and neck squamous carcinoma (HNSCC), and establish a novel metabolism-related prognostic model to explore the clinical potential and predictive value on therapeutic response. We screened 4752 metabolism-related genes (MRGs) and then identified differentially expressed MRGs in HNSCC. A novel 10-MRGs risk model for prognosis was established by the univariate Cox regression analysis and the least absolute shrinkage and selection operator (Lasso) regression analysis, and then verified in both internal and external validation cohort. Kaplan-Meier analysis was employed to explore its prognostic power on the response of conventional therapy. The immune cell infiltration was also evaluated and we used tumor immune dysfunction and exclusion (TIDE) algorithm to estimate potential response of immunotherapy in different risk groups. Nomogram model was constructed to further predict patients’ prognoses. We found the MRGs-related prognostic model showed good prediction performance. Survival analysis indicated that patients suffered obviously poorer survival outcomes in high-risk group (*p* < 0.001). The metabolism-related signature was further confirmed to be the independent prognostic value of HNSCC (HR = 6.387, 95% CI = 3.281-12.432, *p* < 0.001), the efficacy of predictive model was also verified by internal and external validation cohorts. We observed that HNSCC patients would benefit from the application of chemotherapy in the low-risk group (*p* = 0.029). Immunotherapy may be effective for HNSCC patients with high risk score (*p* < 0.01). Furthermore, we established a predictive nomogram model for clinical application with high performance. Our study constructed and validated a promising 10-MRGs signature for monitoring outcome, which may provide potential indicators for metabolic therapy and therapeutic response prediction in HNSCC.

## Introduction

Head and neck squamous carcinoma (HNSCC) is a heterogeneous epithelial tumor, accounting for more than 830,000 newly diagnosed cases, and no more than half a million individuals’ deaths annually in the world (1). Although advances in various treatment regimens, patient’s prognosis is still poor (2). The 5-year survival time is only nearly 50% and there is strong tendency of distant metastasis and local recurrence in HNSCC (3). There are limited effective indicators that can predict prognosis in HNSCC patients (4). It is hence significant to identify novel predictors for clinical outcomes and predicting therapeutic response to provide new insights for optimizing individual treatment in HNSCC.

The oncogenesis and development of HNSCC are closely associated with viral infection, genetic changes, environmental factors and imbalanced metabolism (5). Metabolic reprogramming is regarded as a hallmark of cancers, which defined as the remarkably changes of metabolic patterns in the occurrence and evolution of cancer (6). It includes several metabolic pathways, involving in the glycolysis, amino acid metabolism, the tricarboxylic acid (TCA) cycle, nucleic acid metabolism, etc. (7). It’s significative to reveal the neoplastic metabolic patterns, thereby which may benefit for personalized treatment management and disease monitoring.

Previous findings have shown that the unique metabolic pattern of tumor cells is a positive alteration of heredity expression to regulate the neoplasm oncogenesis and invasiveness (8, 9). Regulation of metabolism-related genes (MRGs) was suggested to be an important mechanism by which the specific metabolic mode exerts its functions in cancers (10). Given the targeting tumor metabolism as a potential and promising novel treatment strategy, exploring MRGs-related signature which can serve as a predictor for the evaluation of diagnosis, treatment, and prognosis, has received increasing attention and demonstrated in several cancers (11–13). Some recent studies also revealed the imbalance metabolic patterns of HNSCC in a single metabolic pathway or metabolite, based on glycolysis (14), lipid metabolism (15, 16), and pyruvate catabolism (17). However, the overall landscape of MRGs in HNSCC has not been fully revealed and the predictive role on therapy response remains unclear.

In present study, we use the public dataset, The Cancer Genome Atlas (TCGA) databases, to develop a MRGs-related risk model in HNSCC. We evaluated and verified the specificity and sensitivity of this model to determine prognostic significance and make an evaluation into its prognostic value on both conventional therapy and immunotherapy in HNSCC.

## Materials and Methods

### Samples and Data Acquisition

The whole-genome RNA-seq data (normalized RPKM) with corresponding somatic mutation data and clinical information was obtained from the TCGA database (https://portal.gdc.cancer.gov/). The somatic mutation data of tumor samples was utilized to calculate tumor mutation burden (TMB), which refers to the frequency of somatic alterations per megabase of the genome (18). Moreover, patients’ clinical follow-up information was collected, including age, gender, grade, TNM stage, survival time (days), survival status, and application of radiotherapy or chemotherapy. An independent HNSCC cohort (GSE41613 from GPL570 platform) with complete overall survival (OS) information was extracted from the Gene Expression Omnibus (GEO) available database (https://www.ncbi.nlm.nih.gov/gds/) for validation (19). We used R package “sva” to remove data batch effects. These data were obtained from the online public sources, so all informed consents were available and we conducted this study as requested on using these databases.

### Identification of MRGs Set

A list of 2752 MRGs was extracted from previously published report, which was associated with metabolism process and encoded the known human metabolic enzymes and transporters (20). Additionally, differentially expressed genes (DEGs) between HNSCC and compared normal samples were derived from the GEPIA v2.0 database (http://gepia2.cancer-pku.cn/), with the criteria of the threshold value of adj. p < 0.01 and |log2 fold change (FC)| > 1.0. Venn was used to identify differentially expressed metabolism-related genes (DEMRGs) by the two groups drawn using “VennDiagram” R packages. We presented a protein-protein interaction (PPI) network *via* the Search Tool for Recurring Instances of Neighbouring Genes (STRING) database (https://string‐db.org/cgi/input.pl) for the overlapping DEMRGs to retrieve interaction.

### Gene-Enrichment and Functional Annotation Analysis

Gene functional enrichment analysis were carried out to illustrate various functions and differential disturbing pathways of DEMRGs, including the Gene Ontology (GO) and Kyoto Encyclopedia of Genes and Genomes (KEGG) enrichment analysis, conducted by “clusterProfiler” R package. GO analysis, consisting of biological processes (BPs), molecular functions (MFs), and cellular components (CCs), offers integrative annotation to identify characteristic biological meaning in MRGs. We applied the “GOplot” R package to visualize the enrichment terms. Additionally, the KEGG analysis revealed potential signaling pathways which the candidate MRGs participate in. The results accurately were visualized by the R packages “enrichplot” and “ggplot2”.

### Construction and Validation of the Risk Model Based on MRGs

Totally 499 HNSCC patients were randomly divided at a 7:3 ratio, served as the training and internal validation cohorts with the “caret” R package. The prognostic value of DEMRGs on HNSCC patients was evaluated by the univariate Cox regression model using the data from the training cohort. Initially, the potential prognostic DEMRGs were selected (*p*-value < 0.01 was chosen). Then the least absolute shrinkage and selection operator (Lasso) regression analysis was screened to construct the MRGs-derived risk signature with the “glmnet” R package. We used CBioPortal (http://www.cbioportal.org/) to show the related genetic changes of particular genes. The formula of risk score was generated as following: risk score = ∑ (expression level of each particular gene × individual regression coefficient). According to the median of risk score as the cutoff, we classified subjects into low- and high-risk groups. PCA was performed to explore the distribution of different groups with the “stats” R package, based on the expression of genes in the signature. Besides, survival differences were assessed by Kaplan-Meier curve using the “survival” R packages. Receiver operating characteristic (ROC) curves were conducted to investigate the prediction accuracy of the model for 3/5-year OS using area under the ROC curve (AUC) estimation with the “survival ROC” R package. An AUC value of 0.70 or greater was considered as a significant predictive value. The internal validation cohort and GSE41613 cohort were also subject to the same analysis to verify the predictive power of the risk signature.

### Correlation Analysis of Clinicopathologic Characteristics

Univariate and multivariate Cox regression analysis were carried out to examine the prognostic significance of the risk score and clinicopathologic characteristics for OS. We further evaluated differences of prognostic MRGs and risk score in clinical features. Subsequently, we employed the “survival” R packages to estimate the clinical utility of risk model for patients subjected to chemotherapy or radiotherapy.

### Tumor-Infiltrating Immune Cell Analysis

In order to characterize immune cell infiltration in tumour microenvironment (TME) in different risk groups, two algorithms, the single sample Gene Set Enrichment Analysis (ssGSEA) algorithm and the CIBERSORT were performed based on the gene expression in TCGA training cohort. The former calculated HNSCC tissue purity, immune and stromal scores using the “ESTIMATE” R package. The relative fractions of 22 types of immune cell in TME in each sample of HNSCC were determined by “CIBERSORT” R package with all proportions normalized to 1 in each sample. This algorithm used Monte Carlo sampling to calculate a p-value for each case, and cases with *p*-value > 0.05 were excluded for subsequent analysis. We illustrated the results as a stacked bar plot, with the various immune cells. The box plots were exhibited for better visualization to show the difference abundance of infiltrating immune cells with “ggplot2” R package. Subsequently, we evaluate the correlations of immune cell infiltration and risk score.

### Prediction of Immune Checkpoint Blockade (ICB) Therapy Response

Potential tumor therapeutic response with ICB was predicted by Tumor Immune Dysfunction and Exclusion (TIDE) score, a computational algorithm based on gene expression profile (http://tide.dfci.harvard.edu). It demonstrated that patients exhibit lower sensitivity with ICB agents, who had high TIDE score, thus indicating high possibility of antitumor immune escape (21). The details of the algorithm are described elsewhere (21). Meanwhile, the TMB, also as a useful marker for ICB selection, was performed to validate the predictive effectiveness of immunotherapy response.

### Construction and Validation of the Nomogram Model

We established a nomogram model using prognosis factors from univariate Cox regression analysis to visualize the score for each variable on the point scale by the “rms” R package. Then we evaluated the predictive probabilities for 3- and 5-year clinical outcomes by depicted calibration curves. The ROC curves were also employed to determine the exactitude of the model.

### Statistical Analysis

R software v3.6.2 (https://www.r-project.org/) together with SPSS Statistics v25.0 assisted in performing all statistical analysis. We also used R software, GraphPad Prism 7.0 and Cytoscape software for diagram drawing. Unpaired Student’s *t*-test and Whitney *U*-test were employed for comparing differences between two sets containing parameters with normally and non-normally distributions, respectively. A Chi-square test was used for categorical variables. A Wilcoxon rank-sum test was applied to compare non-parametric parameters for two sets, while the Kruskal-Wallis test was used for multiple sets. Pearson’s chi-square test was made to analyze the correlation between qualitative parameters. If not specified above, it represented statistical significance with *p*-value < 0.05.

## Results

### Initial Screening of DEMRGs in HNSCC From the TCGA Database

**Figure 1:**
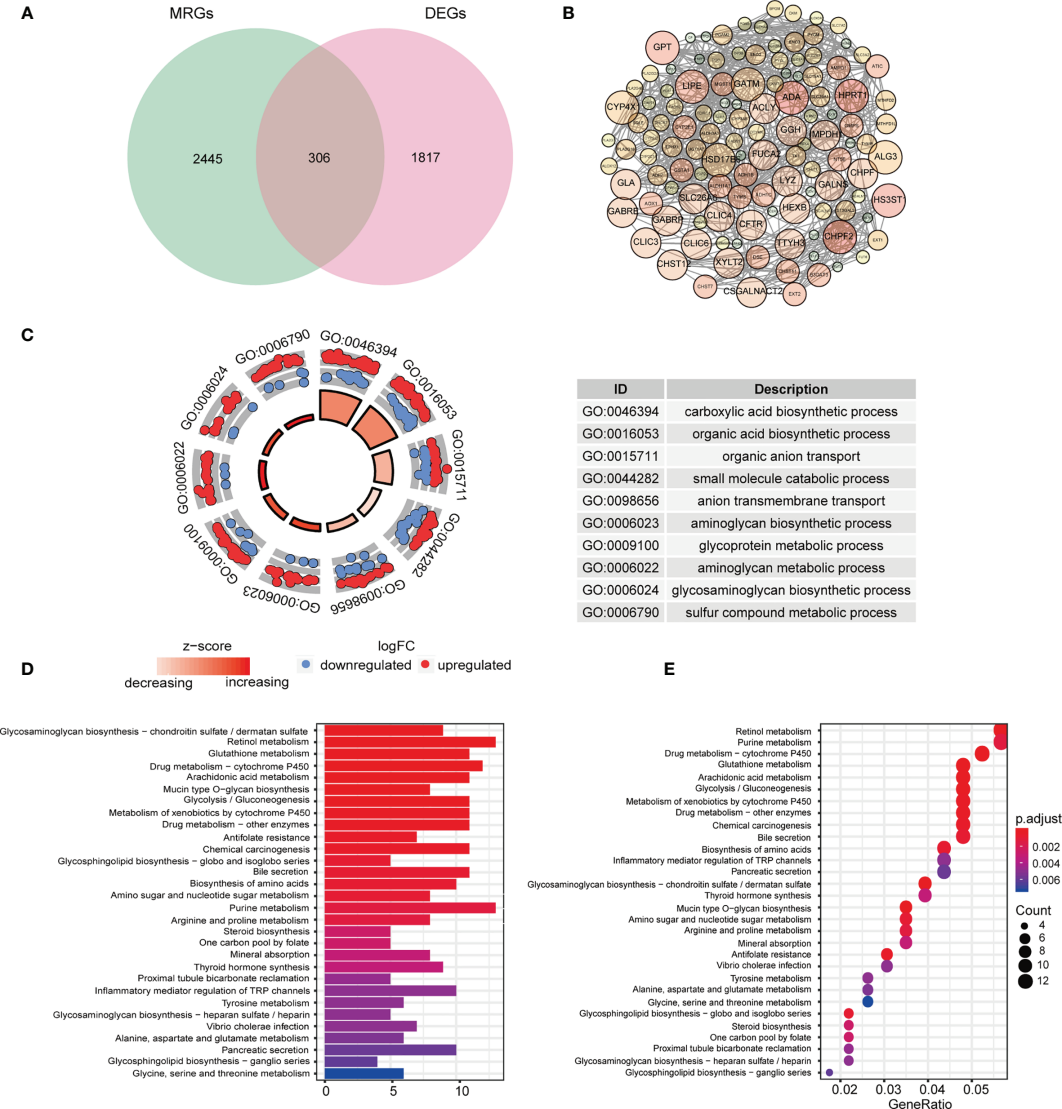
Identification and functional analysis of candidate DEMRGs. **(A)** Venn diagram depicting overlap to identify DEMRGs between HNSCC tumor and normal tissue. **(B)** The interactions among MRGs showing in PPI network. **(C)** The GO circle plot of biological process (BP) differentially enriched in patients. **(D, E)** Enriched KEGG pathways of differentially expressed MRGs.

The HNSCC transcriptome data comprised 546 samples, which involved 502 tumor samples and 44 normal samples from TCGA database. Among 2752 genes involved in metabolism, 2751 MRGs were finally selected after filtering repeated genes. Afterward, we analyzed the DEGs between cancer and compared normal samples from online database and 2751 MRGs, and then identified 306 DEMRGs shown in **Figure 1A**. We identified PPI network of DEMRGs with high confidence levels (> 0.7) in **Figure 1B**. After excluding the samples without complete survival information, 499 HNSCC patients were finally enrolled for further analysis.

### Gene-Enrichment and Functional Annotation for DEMRGs in HNSCC

According to the functional enrichment analysis of the 306 DEMRGs, we obtained GO and KEGG annotations of these genes in HNSCC. Not surprisingly, it showed that these genes were significantly related to metabolism process, including organic compounds biosynthesis, transport and metabolic process in the biological processes (**Figure 1C**). In the CC term of GO analysis, membrane-transport-related components, such as “intrinsic component of Golgi membrane”, “transporter complex”, and “integrin complex” were significantly enriched by DEMRGs (**Figure S1**). In terms of MF analysis, “transmembrane transporter activity”, “transferase activity” and “oxidoreductase activity” were mainly categories for these genes to play important roles (**Figure S1**). For KEGG analysis, these DEGs were also shown to mainly enrich in several tumor metabolic pathways, like amino acids, purine and drug metabolism. Interestingly, it indicated that these genes were also related to chemical carcinogenesis as general oncogenes (**Figures 1D, E** **)**.

### Construction and Internal Validation of the MRGs-Related Prognostic Risk Signature

**Figure 2:**
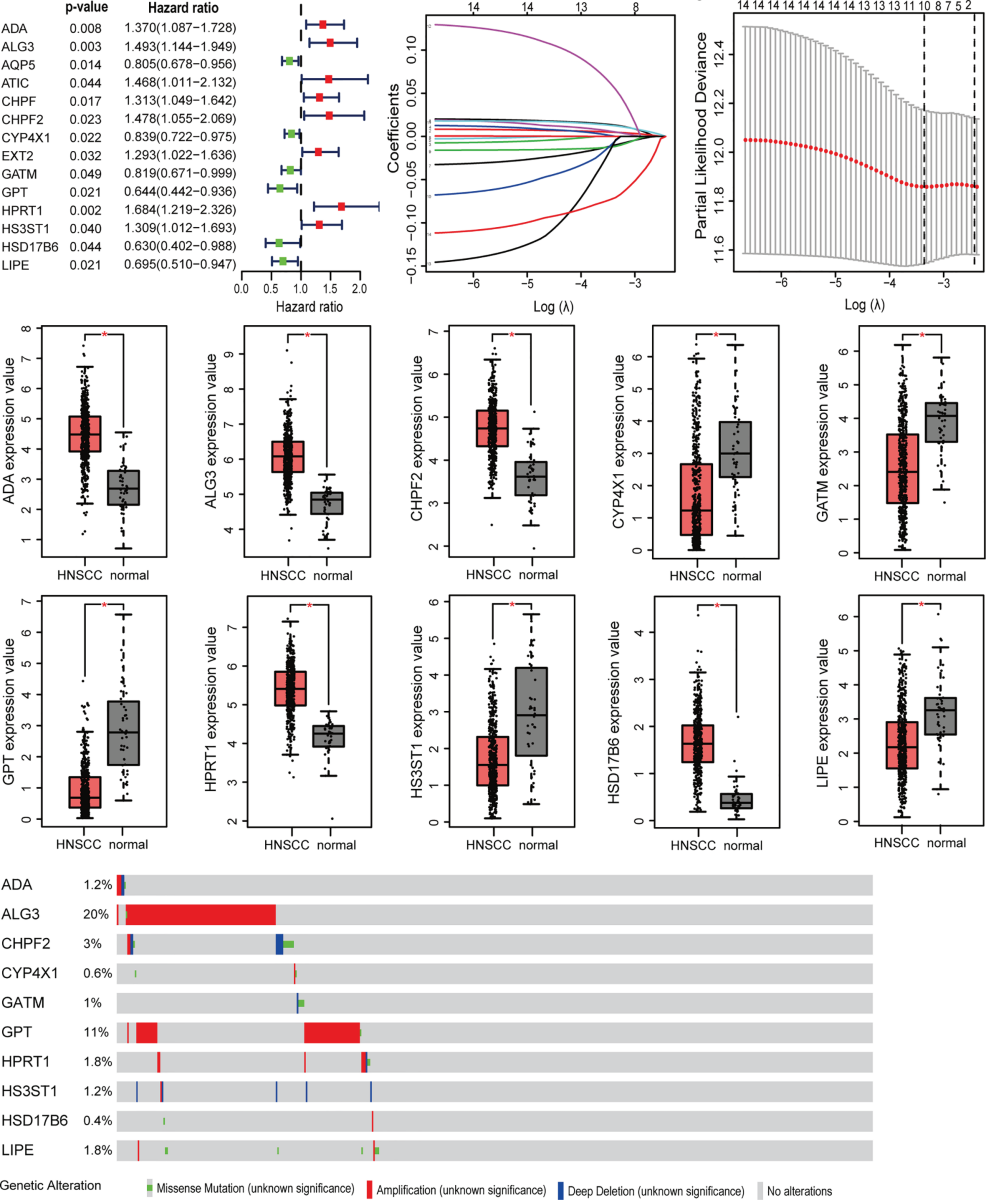
Prognosis MRGs-related signature identified by Lasso regression analysis. **(A)** Forest plots showing the prognostic metabolic genes determined by the univariate Cox regression analysis (green represents protective factors, and red represents risk factors). **(B)** Lasso coefficient profiles of selected 14 MRGs and **(C)** the optimal values of the penalty parameter λ for OS. **(D)** The expression of 10 MRGs based on TCGA databases from GEPIA. **(E)** The mutation patterns of 10 differentially expressed MRGs. (*p < 0.05).

499 HNSCC patients were divided into two cohorts, namely, a training set with 350 patients and an internal validation set with 149 patients. **Table 1** presented the baseline clinical characteristics, which showing the compared characteristics of the two cohorts were matched. Next, the univariate Cox regression analysis was performed to identify prognosis-related genes using 306 DEMRGs in the training group. A total of 14 DEARGs were obviously associated with the OS (**Figure 2A**, *ps*< 0.05). To construct an optimal risk model for predicting prognosis of HNSCC patients, 10 MRGs identified by Lasso regression analysis were used for further research on their prognostic value (**Figures 2B, C** **)**. Among these 10 genes, ADA, ALG3, CHPF2, HPRT1, and HSD17B6 showed higher expressions than adjacent non-cancerous tissues, while CYP4X1, GATM, GPT, HS3ST1, and LIPE were low expressions in tumors (**Figure 2D**). The mutational landscape of 10 MRGs revealed ALG3 was the most frequently mutated gene (20%), followed by GPT with an alteration frequency of 10% (**Figure 2E**). The detailed interactions of these 10 genes in MRGs were presented in **Figure S2**. The coefficients to calculate risk scores based on expression level of genes obtained by the LASSO algorithm were listed in **Table S1**.

**Table 1:** Demographic characteristics of the training cohort and the test cohort in HNSCC.

		Training cohort (N = 350)	Validation cohort (N = 149)	*P* Value
Age	≤65	227 (64.9%)	97 (65.1%)	0.958
	>65	123 (35.1%)	52 (34.9%)	
Gender	Male	250 (71.4%)	116 (77.9%)	0.137
	Female	100 (28.6%)	33 (22.1%)	
Grade	G1-2	258 (73.7%)	101 (67.8%)	0.124
	G3-4	78 (22.3%)	43 (28.9%)	
	NA	14 (4.0%)	5 (3.4%)	
Stage	Stage I-II	69 (19.7%)	36 (24.1%)	0.265
	Stage III-IV	281 (80.3%)	113 (75.9%)	
T	T1-2	123 (35.1%)	53 (35.5%)	0.768
	T3-4	222 (63.4%)	90 (60.4%)	
	NA	5 (1.4%)	6 (4.0%)	
N	N0	177 (50.6%)	63 (42.3%)	0.110
	N1-3	161 (46.0%)	79 (53.0%)	
	NA	12 (3.4%)	7 (4.7%)	
M	M0	332 (94.9%)	142 (94.0%)	0.145
	M1	5 (1.4%)	0 (0.0%)	
	NA	13 (3.7%)	7 (4.7%)	

NA, not applicable; Ambiguous variables (NA) were excluded from Chi-square test.

**Figure 3:**
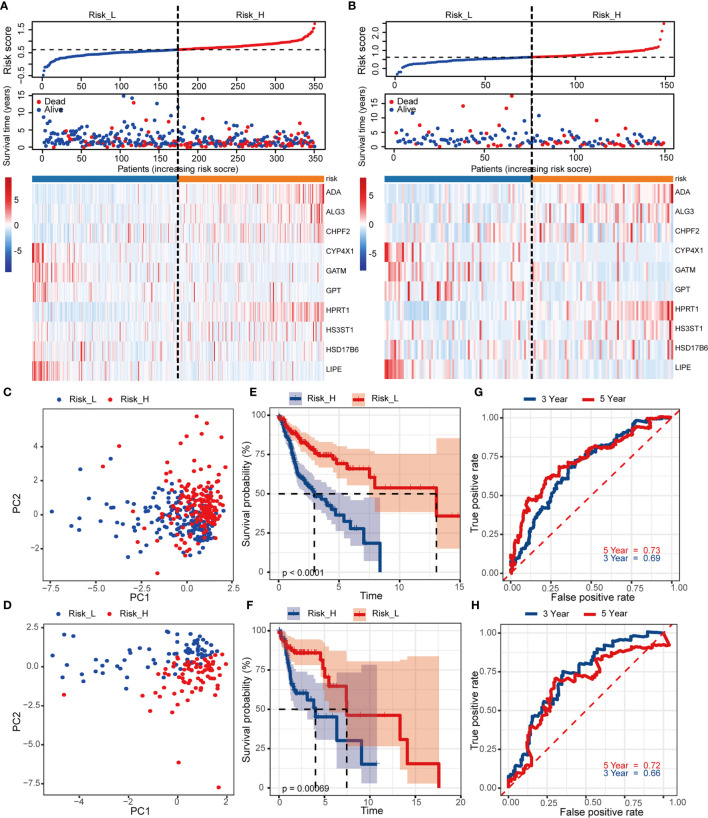
Construction and internal validation of the prognosis-related risk model based on MRGs in TCGA cohort. The risk scores distribution, vital status of patients, and heatmap of identified 10 MRGs expression level in **(A)** training and **(B)** validation cohorts. **(C, D)** PCA plot of prognostic metabolism signature. **(E, F)** Kaplan-Meier curves of OS and **(G, H)** time-dependent ROC analysis for 3/5-year survival to predict OS.

To detect the robustness of the model established by training set, we utilized the same coefficients to calculate the risk scores in validation cohort. Based on the median value of risk score (0.63) as a cut-off point, subjects were classified into high- and low-risk groups in training and validation sets, respectively. **Figures 3A, B** exhibited the ranked risk scores’ distribution, survival status of individual patients, and the heatmap of 10 MRGs expression in two cohorts. The distribution map of survival status showed patients suffered more mortal risks with increasing risk score. The heatmap results suggested that therisky MGRs, including ADA, ALG3, CHPF2, HPRT1, and HS3ST1, were highly expressed in the high-risk group, while the expression of CYP4X1, GATM, GPT, HSD17B6, and LIPE were upregulated as protective MRGs in the low-risk group. PCA analysis depicted by scatter plots revealed the patterns of risk-genes expression in different risk groups (**Figures 3C, D** **)**. The Kaplan-Meier curves showed remarkably worse OS with high risk (**Figures 3E, F**, *p* < 0.001) in training and validation cohort. To evaluate the predictive power of the risk score, the time-dependent ROC curves were utilized and the AUC for 3-, and 5-year OS reached 0.69, and 0.73, respectively (**Figure 3G** **)**. Consistently, as shown in **Figure 3H**, the AUC values for 10 risk signatures were 0.66 at 3 year, and 0.72 at 5 years in validation cohorts.

### Validation of the MRGs-Related Prognostic Risk Model *via* External Cohort

**Figure 4:**
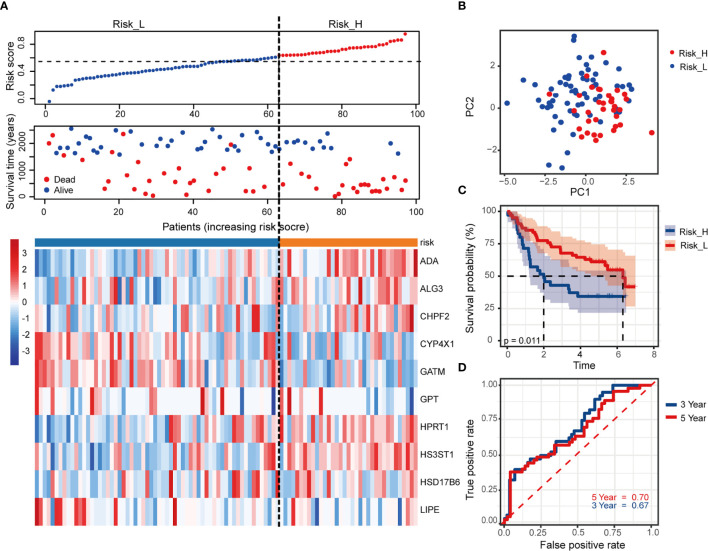
Validation of the risk signature in external GSE41613 cohort. **(A)** The distribution of risk score, survival status of patients and heatmap of 10 MRGs. **(B)** PCA plot of prognostic metabolism signature. **(C)** Kaplan-Meier curves of OS and **(D)** time-dependent ROC curve of 3- and 5-year survival rate.

We used an independent cohort with 97 patients from GEO dataset GSE41613 to verify the model accuracy externally. The risk scores and grouping did with the same ways mentioned previously (the median value of risk score = 0.63). **Figure 4A** showed the survival status together with the 10 MRGs expression, based on patients with increasing risk scores. PCA analysis showed a segregated tendency of gene expression patterns in two risk groups based on MRGs-related signature (**Figure 4B**). As demonstrated by TCGA cohorts, patients with high risk exhibited worse OS (**Figure 4C**, *p* = 0.011). Correspondingly, the 3- and 5-year AUC value in predicting prognosis were 0.67 and 0.70, respectively (**Figure 4D**). It was further revealed the predictive performance of the 10-MRGs prognosis signature of HNSCC patients by these results.

### Assessment of Relevance Between the Prognostic Signature and Clinical Characteristics

**Figure 5:**
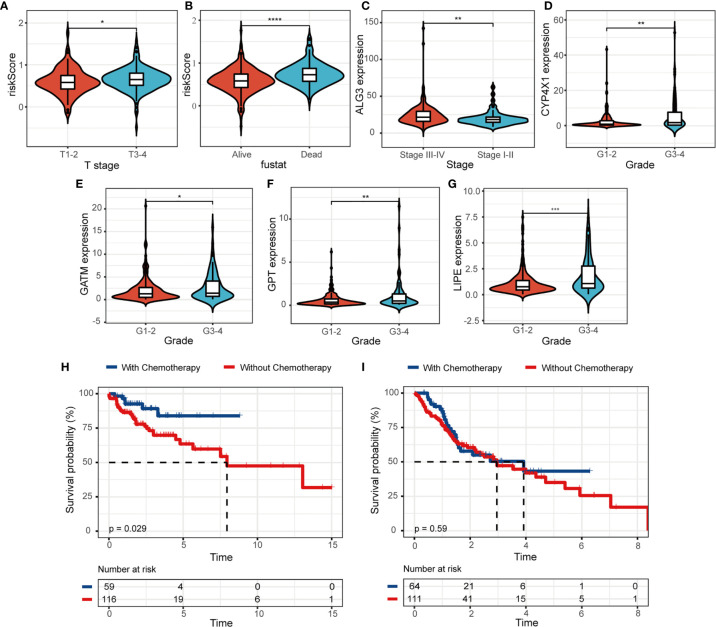
The relevance between the risk model and clinicopathologic features. **(A, B)** The correlations between risk score and clinical characteristics. **(C–G)** The associations between MRGs expression level and clinical features. Kaplan-Meier survival analysis for patients in **(H)** low- and **(I)** high-risk groups with the application of chemotherapy. (*p < 0.05; **p < 0.01; ***p < 0.001; ****p < 0.0001).

Univariate and multivariate Cox regression analysis were utilized to explore prognostic factors for OS in TCGA cohorts, as shown in **Table 2**. Among the available variables, age, gender, stage and risk score (HR = 6.945, 95% CI = 3.538-13.632, *p* < 0.001) were prognosis factors of HNSCC in the training cohort. However, it was validated that only the risk score was significantly correlative with OS *via* validation cohort (HR= 3.217, 95% CI = 1.691-6.118, *p* < 0.001). The results of multivariate Cox regression analysis indicated the role of risk score working as an independent predictor for HNSCC prognosis (training cohort: HR =6.387, 95% CI = 3.281-12.432, *p* < 0.001; validation cohort: HR=2.812, 95% CI = 1.439-5.495, *p* = 0.002). Then we further evaluated the associations between clinical features and risk signature. We found that the risk score was correlated with survival state, as mentioned above (**Figure 5A** **)**. And the T3-4 stage had higher risk scores than T1-2 stage (*p* < 0.05), indicting poor prognosis, as shown in **Figure 5B**. In addition, some signature-related genes were differentially expressed among clinical parameters. Patients with stage III-IV had higher ALG3 expression than early stage (**Figure 5C** **)**. And the expression of CYP4X1, GATM, GPT, and LIPE were related to grade (**Figures 5D–G**).

**Table 2:** Univariate and multivariate Cox regression analyses of OS in the TCGA training and internal validation cohorts of HNSCC.

Variables	Univariate analysis		Multivariate analysis	
	HR (95% CI)	*p*-value	HR (95% CI)	*p*-value
TCGA training cohort				
Age	1.033 (1.015−1.051)	**<0.001**	1.031 (1.012−1.051)	**0.002**
Gender	0.622 (0.423−0.914)	**0.016**	0.833 (0.548−1.266)	0.393
Grade	0.985 (0.731−1.328)	0.923	1.081 (0.797−1.467)	0.617
Stage	1.326 (1.050−1.674)	**0.018**	1.681 (1.237−2.283)	**<0.001**
T	1.025 (0.843−1.247)	0.803	0.801 (0.635−1.009)	0.06
N	1.055 (0.851−1.306)	0.626	1.052 (0.830−1.334)	0.674
Risk score	6.945 (3.538−13.632)	**<0.001**	6.387 (3.281−12.432)	**<0.001**
TCGA validation cohort				
Age	1.007 (0.980−1.034)	0.622	1.004 (0.975−1.035)	0.785
Gender	1.855 (0.823−4.181)	0.136	1.589 (0.684−3.690)	0.281
Grade	1.353 (0.877−2.090)	0.172	1.151 (0.725−1.826)	0.551
Stage	1.198 (0.850−1.690)	0.302	1.170 (0.684−2.002)	0.566
T	1.123 (0.811−1.555)	0.484	0.999 (0.628−1.591)	0.997
N	1.066 (0.774−1.470)	0.694	0.964 (0.647−1.436)	0.856
Risk score	3.217 (1.691−6.118)	**<0.001**	2.812 (1.439−5.495)	**0.002**

HR, Hazard ratio. Bold value showed significance.

We further evaluated the clinical utility of risk model for patients subjected to radiotherapy or chemotherapy in HNSCC. We observed that HNSCC patients would benefit from the application of chemotherapy, patients with chemotherapy (n = 59) had a higher probability of survival than those without chemotherapy (n = 116) in the low-risk group (**Figure 5H**, *p* = 0.029). The survival benefit with chemotherapy was not significant in the high-risk group (n = 64, with chemotherapy; n = 111, without chemotherapy; *p* = 0.59, **Figure 5I**). We didn’t observe the same results in the application of radiotherapy (**Figures S3A, B**). Moreover, patients with low risk score significantly suffered more beneficial prognosis from chemotherapy (**Figure S3C**, *p* < 0.001) or radiotherapy (**Figure S3D**, *p* = 0.012).

### Comprehensive Analysis of Immune Characteristics and ICB Therapy in Different Risk Subtypes

**Figure 6:**
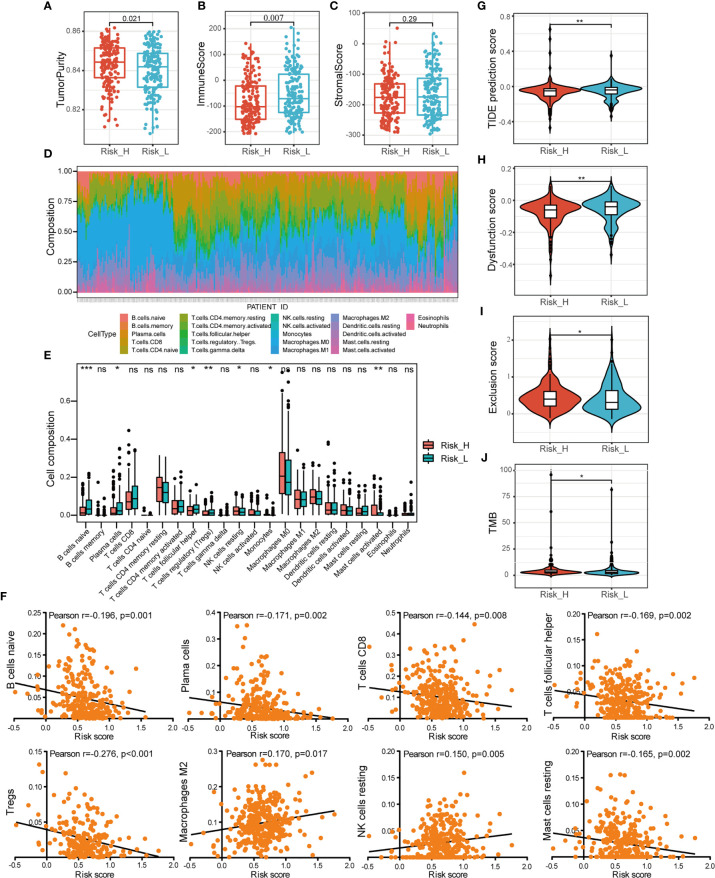
Comprehensive analysis of immune characteristics. **(A–C)** Comparison of the ssGSEA scores (tumor purity, immune and stromal scores) between different risk groups. **(D)** A stacked bar plot presenting the fractions of 22 types of infiltrating immune cell estimated using CIBERSORT algorithm in HNSCC from TCGA training cohort. **(E)** The differences of immune cells infiltration between high- and low-risk groups in boxplots. **(F)** The correlations between immune cells infiltration and risk scores. **(G–I)** The violin plot of different prediction score of TIDE, together with dysfunction score and exclusion score in two groups analyzed by the t-test. **(J)** The difference of TMB in two groups. (ns, *p* > 0.05, *p < 0.05; **p < 0.01; ***p < 0.001).

To elucidate the difference of immune microenvironment of HNSCC, we analyzed the immune scores and immune cell infiltrating density between high- and low-risk groups. It revealed significant differences in purity of tumor (*p* =0.021, **Figure 6A**) and immune scores (*p* = 0.007, **Figure 6B**), but not in stromal scores (**Figure 6C**) between the two groups. The high-risk group showing lower immune scores presented lower immune infiltration in TME. It also indicated poor prognosis which was consistent with worse survival outcomes of patients in the high-risk group. Subsequently, we analyzed the fraction of 22 types of immune cell *via* CIBERSORTx algorithms. The stacked bar plot illustrated the infiltrating difference of immune cells in each case (**Figure 6D**). Additionally, **Figure 6E** showed the differential distributions of infiltrating immune cells in the two risk groups. Among them, the infiltration levels of naive B cells (*p* < 0.001), plasma cells (*p* < 0.05), T cell follicular helpers (Tfh) (*p* < 0.05), and regulatory T cells (Tregs) (*p* < 0.01) were significantly higher in low-risk group. While the resting NK cells (*p* < 0.05), monocytes (*p* < 0.05), and activated mast cells infiltration (*p* < 0.01) were higher in high-risk group. Moreover, we evaluated the relationships between different immune infiltrates and risk score. The infiltration of naive B cells, plasma cells, CT8+ T cells, Tfh, Tregs, and resting mast cells was found to be negatively associated with prognostic risk, while M2 macrophages and resting NK cells were positively associated (**Figure 6F**).

To investigate the association between risk subtyping of HNSCC and its response of immune checkpoint inhibitor, we estimated the potential clinical response of ICB therapy with TIDE algorithm. The low-risk group was featured by higher TIDE score (*p* < 0.01, **Figure 6G**), composed of higher TIDE dysfunction signatures (*p* < 0.01, **Figure 6H**) and lower TIDE exclusion scores (*p* < 0.05, **Figure 6I**), compared to the high-risk group. It indicated the mechanism of tumor immune evasion was mainly associated with the infiltration of dysfunctional tumor cytotoxic T lymphocytes (CTL) in low-risk group. What’s more, the patients with high risk score were featured by higher TMB shown in **Figure 6J** (*p* < 0.05). Our findings suggested that ICB therapy may be effective for HNSCC patients with high-risk subtype.

### Construction and Validation of the Nomogram Model

**Figure 7:**
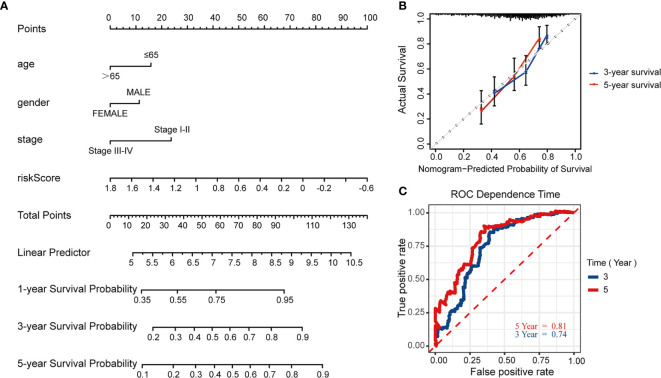
Construction and validation of the predictive nomogram model. **(A)** Nomogram model established by prognosis factors to estimate the survival possibility for 3 and 5 years in HNSCC patients. **(B)** The calibration curves for validation of the nomogram. **(C)** The time-dependent ROC curves of the nomogram of 3-year and 5-year survival rate.

To further predict patients’ prognoses, we constructed a nomogram that enrolled all prognosis factors, including age, gender, TNM stage, and risk score to quantitatively predict HNSCC prognosis in the TCGA training cohort (**Figure 7A**). It was an assessment that is tailored to an individual HNSCC patient, the estimate probability of 3- and 5-year OS was easy to ascertain by calculating each score for prognosis variables and then gaining the total score on the point scale. Compared to the other prognosis factors, risk score based on MRGs-related signature had the most score points. Calibration curves were developed to validate the nomogram’s performance. It showed that the curves were close to the 45° line which was regarded the best prediction (**Figure 7B**). Meanwhile, the AUC for the nomogram model was 0.74 and 0.81, respectively (3‐, 5‐year OS prediction), which significantly improved the predictive performance, compared to the AUC of risk signature (**Figure 7C**).

## Discussion

In this study, we used mRNA expression data together with 4751 MRGs to analyze the overall landscape of imbalance metabolism in HNSCC. Functional analysis confirmed differentially expressed MRGs primarily involved in amino acids, purine and drug metabolism process. We constructed a prognosis-related 10-MRGs signature (ADA, ALG3, CHPF2, CYP4X1, GATM, GPT, HPRT1, HS3ST1, HSD17B6, and LIPE) for establishing risk stratification and predicting clinical outcomes. We found lower risk score suggested better survival outcome, which was also effectively validated by internal and external validation cohorts. The metabolism-related risk score was verified to be an independent prognostic factor of HNSCC. The risk signature was associated with survival status and tumor size. Furthermore, we evaluated the clinical role of risk model for predicting responses to various therapies. We observed that patients in low-risk group were more likely to benefit from the application of chemotherapy. ICB therapy may be efficacious for HNSCC patients in high-risk group. Moreover, we constructed a valuable predictive nomogram model with MRGs-related signature and other prognosis factors, which could work as a better predictive model for survival in clinically application.

The role of most of the 10 identified MRGs in oncogenesis and progression of tumors has been reported. ADA, encoding an enzyme involving the crucial purine metabolism, mainly exerted its function on T lymphocytes proliferation and differentiation (22). Besides, some studies also shown the functionality of HPRT in purine regulation (23). It has been revealed that ADA was served as a potential therapeutic target for breast cancer and a biomarker in oral cancer (24, 25). Increased expression of ALG3 was observed in HNSCC cell line (26). Moreover, ALG3 as a class of glycosyltransferases promotes the proliferation and radiosensitivity of cancer cells and is related with poor prognosis and lymph node metastasis (27, 28). CHPF2 acts as an oncogene that is up-regulated in cancers to exert its positive role in cell proliferation and metastasis, including breast cancer, hepatocellular carcinoma, lung adenocarcinoma, *etc.* (29–31). CYP4X1, a member of the cytochromes P450 family, oxidatively metabolizes a wide range of physiological substrates and plays a potential role in the body in response to cancer chemotherapy (32). Additionally, GATM enhances creatine synthesis in relation to the metabolism of human thermogenic fat (33). GPT is thought to be involved amino acid metabolism and be a novel target for cancer therapy (34). HS3ST1 might acts as a molecular biomarker for tumor diagnosis and prognosis (35). HSD17B6 has important functionality in androgen catabolism and was remarkably downregulated in hepatocellular carcinoma, which predicted poor prognosis and high tumor immune infiltrates (36). It was identified that LIPE is up-regulated in cervical cancer and promotes cell proliferation, invasion, and epithelial-mesenchymal transformation (37).

Metabolic reprogramming contributes to patient prognosis, as previous studies demonstrated (9, 38). Recent findings have shown that gene mutations and epigenetic regulation could be triggered by dysfunction metabolism during HNSCC progression (11, 39). Several metabolism-related prognostic biomarkers have been proposed based on gene expression profiles in HNSCC using RNA-Seq technology. Liu and his team developed a new risk model with identified glycolysis-related genes to predict prognosis of HNSCC patients (14). Xiong et al. found lipid metabolism was correlated with worse prognosis and may reflect the immune status of HNSCC patients (15). It was determined that pyruvate catabolism key genes made great progress in HNSCC neoplastic progression (17). Wu et al. provided support for dysregulated metabolism underlying OSCC tumorigenesis (10). Nevertheless, most studies mainly focused on an individual metabolic pathway, which may not sufficiently characterize integrative hallmarks of cancer-related metabolism. In previous study, a metabolism-related signature was identified by several dysregulated metabolic pathways in HNSCC rather than using the common methods based on gene sets, giving the chance to know the impact of abnormal cancer metabolic profiles on survival outcome (40). A comprehensive signature based on multiple MRGs can be better to capture tumorous metabolic changes and have favorable advantage in prognostic prediction (10). Our results also suggested that the risk stratification based on integrative MRGs sets played an important part in HNSCC prognosis and we established a nomogram model with satisfactory predictive value in HNSCC.

HNSCC is a highly heterogeneous malignant tumor with surgery, radiotherapy, chemotherapy, or targeted therapy as single or combinational therapeutic regimens generally (41). In addition, immunotherapy as research hotspot has gained much interest for cancer treatment in recent years. However, only about 20% of patients can benefit from it (42). Accordingly, identification of effective markers contributing to predicting therapeutic response among patients could allow individualization of therapy for HNSCC patients (43, 44). In present study, we found MRGs-related signature could be regarded as an indicator for the efficiency of chemotherapy. However, the survival benefit of HNSCC patients with radiotherapy was to no avail, with probably reason for too much missing clinical information. Compared with conventional therapies, ICB therapy, such as anti-programmed death 1 (PD1) therapy, has been confirmed to obtained indications for treating recurrent or metastatic HNSCC patients in 2016 (42). However, the low response rate becomes the major limitation for survival benefit in patients (41). Deeper characterization of the TME of HNSCC was needed to evaluate the potential predictive role of MRGs signature in tumor immunotherapy.

The immune cell infiltration in the TME plays important roles in the biological behaviors of HNSCC (45). We found patients with superior prognosis and low risk score had higher immune score by ssGSEA algorithm, as demonstrated in several previous studies (46, 47). Additionally, we characterized 22 immune cells infiltration in HNSCC by CIBERSORT algorithm. The infiltration of naive B cells accounted for the most obvious difference in two risk groups, emphasizing their roles in HNSCC. We found the abundance of Tfh, Tregs, and plasma cells were higher in the low-risk group, consisting with the results of Jiang (48). The negative correlation has been recognized between an increasing infiltration of these three cells and a decreased risk score in present study. However, the low-risk group exhibited a higher Tregs infiltration level, which contradicts several previous studies (46, 49, 50). Interestingly, it was found that the monocytes was highly infiltrated in high-risk group, suggesting the HNSCC promoting effect of monocytes in TME (51). What’s more, our results further confirmed the speculation from Jin et al. that resting mast cells may inhibit HNSCC malignant progression (49). As a subgroup of T cells, it was demonstrated that CD8+ T cell infiltration indicated better survival outcomes in HNSCC patients (52). Although we didn’t observe the difference of CD8+ T cells, the correlation analysis revealed the potential tendency of high infiltration in low-risk group. These findings suggested that infiltrated immune cells may occur complicated communications in different metabolism pattern of HNSCC.

We further explored the predictive performance of MRGs-related risk model in the response of immunotherapy. The potential clinical utility of ICB therapy was reflected by TIDE algorithm, which integrates two mechanisms modeling tumor immune evasion, including the CTL dysfunction and exclusion based on gene expression signatures (21). Our study revealed the low-risk group had higher TIDE suggesting poor responsive to ICB therapy with the mechanism of tumor immune escape contributing to high level infiltration of dysfunctional CTL. The dysfunction signatures of TIDE represent the dysfunctional profiles of late-stage T cell (53). Anti-PD1 therapy can recover the function of the early-stage T cells dysfunction, while the dysfunctional T cells at the late stage are resistant to ICB reprogramming (53). Tumour-infiltrating Tregs were reported to exert their immunosuppressive function which contributing to the failure of anti-PD1 immunotherapy (54), which may explain patients with low risk score had poor response to ICB with high Tregs infiltration. Additionally, investigations have revealed that TMB can trigger T-cell responses and neoantigen-rich tumors may be responding to immunotherapy (55, 56). Compared to overexpressed PD-L1, as a prognostic biomarker in tumor cells, high TMB is regarded as a predictive indicator implicated a greater potential of immunotherapy response (57). The high-risk group in this study had significantly higher TMB, which result in exposing neoantigens continuously and subsequent activating the immune response (56). It further supported our result that patients with high risk score may exhibit a better therapeutic response to ICB.

Despite we constructed a prognostic signature and provide innovative insights for improving HNSCC management, there still exist several limitations in present study. On one hand, the missing data may affect the results to a certain degree, which resulting in the lack of prognostic values of radiotherapy. And our external validation cohort only consisted of OS, which cannot further validate our signature effectively. The findings need to be validated by more independent cohorts to prove the clinical utility of risk model. Furthermore, further functional experiments require to explore the underlying mechanisms of 10 MRGs in HNSCC. Finally, the links between the immune activity and risk score have not yet been experimentally confirmed. A prospective clinical trial is encouraged to further validate the clinical value of risk model in ICB decision.

## Conclusion

In conclusion, we constructed a novel 10-metabolic-genes signature and confirmed their prognostic value in HNSCC. Our study also provides potential promising indicators for predicting therapeutic response, which contributing to HNSCC individualized treatment in clinical practice.

## Data Availability Statement

Publicly available datasets were analyzed in this study. This data can be found here: https://portal.gdc.cancer.gov/ https://www.ncbi.nlm.nih.gov/gds/.

## Author Contributions

Conceptualization and writing: YL. Methodology: YW and YP. Software: ZH and XC. Validation: WH. Investigation: TL. Data curation: LW, Formal analysis: WL. Project administration and funding acquisition: SQ. All authors contributed to the article and approved the submitted version.

## Funding

The project was funded by the grants of Joint Funds for the innovation of science and Technology, Fujian province (2018Y9105); United Fujian Provincial Health and Education Project for Tackling the Key Research, China (2019-WJ-03); Science and Technology Program of Fujian Province, China (2018Y2003); National Natural Science Foundation of China (11974077); and National Natural Science Foundation of China (82072986).

## Conflict of Interest

The authors declare that the research was conducted in the absence of any commercial or financial relationships that could be construed as a potential conflict of interest.

## Abbreviations

HNSCC, head and neck squamous carcinoma; MRGs, metabolism-related genes; TCGA, The Cancer Genome Atlas; TMB, tumor mutation burden; GEO, Gene Expression Omnibus; DEGs, differentially expressed genes; DEMRGs, differentially expressed metabolism-related genes; PPI, protein-protein interaction; STRING, Search Tool for Recurring Instances of Neighbouring Genes; GO, Gene Ontology; KEGG, Kyoto Encyclopedia of Genes and Genomes; Lasso, least absolute shrinkage and selection operator; ROC, Receiver operating characteristic curve; AUC, area under the curve; TME, tumour microenvironment; ssGSEA, single sample Gene Set Enrichment Analysis; ICB, immune checkpoint blockade; TIDE, Tumor Immune Dysfunction and Exclusion; Tfh, T cell follicular helpers; Tregs, regulatory T cells; CTL, cytotoxic T lymphocytes.
