# Supplementary material for: A Novel Prognostic Signature Based on Metabolism-Related Genes to Predict Survival and Guide Personalized Treatment for Head and Neck Squamous Carcinoma
Source: Front Oncol. 2021 Jun 14;11:685026. doi: 10.3389/fonc.2021.685026 (PMC8236898; doi:10.3389/fonc.2021.685026)
Supplement: Supplementary file 1 [file DataSheet_1.docx]

Supplementary Material

# Supplementary Figures and Tables

## Supplementary Figures


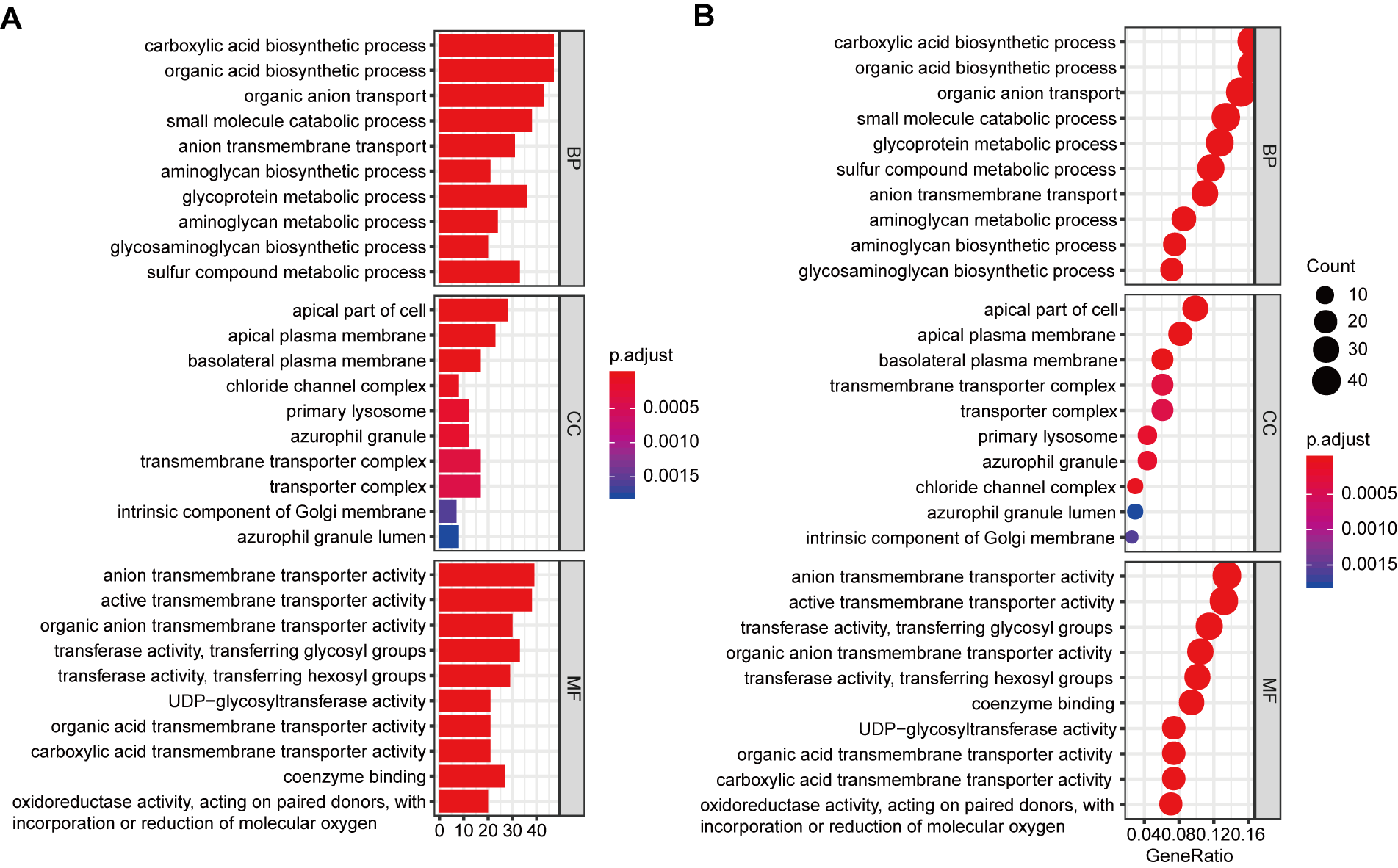


**Supplementary Figure 1.** GO analysis of differentially expressed MRGs. (A) Significantly enriched biological process (BP), cellular components (CC), and molecular functions (MF) terms of DEMRGs. (B) The highly enriched pathways in three terms.


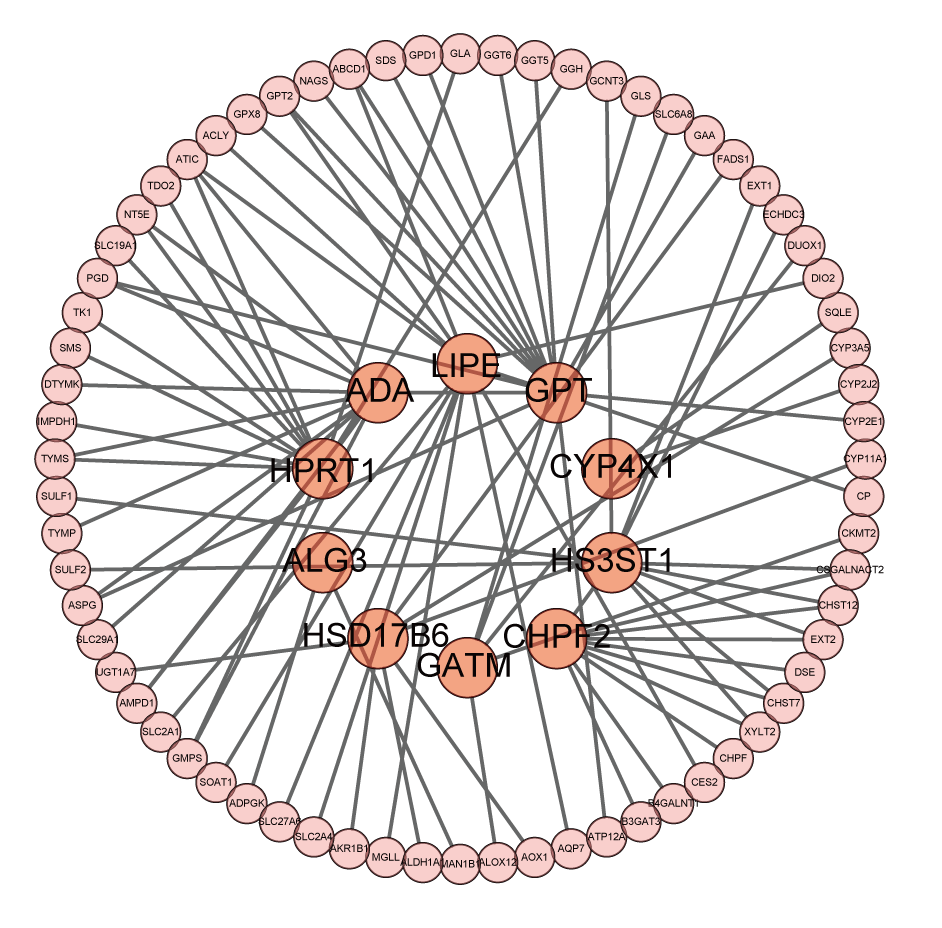


**Supplementary Figure 2**. The interactions of prognosis-related 10 risk signature among MRGs showing in PPI network.


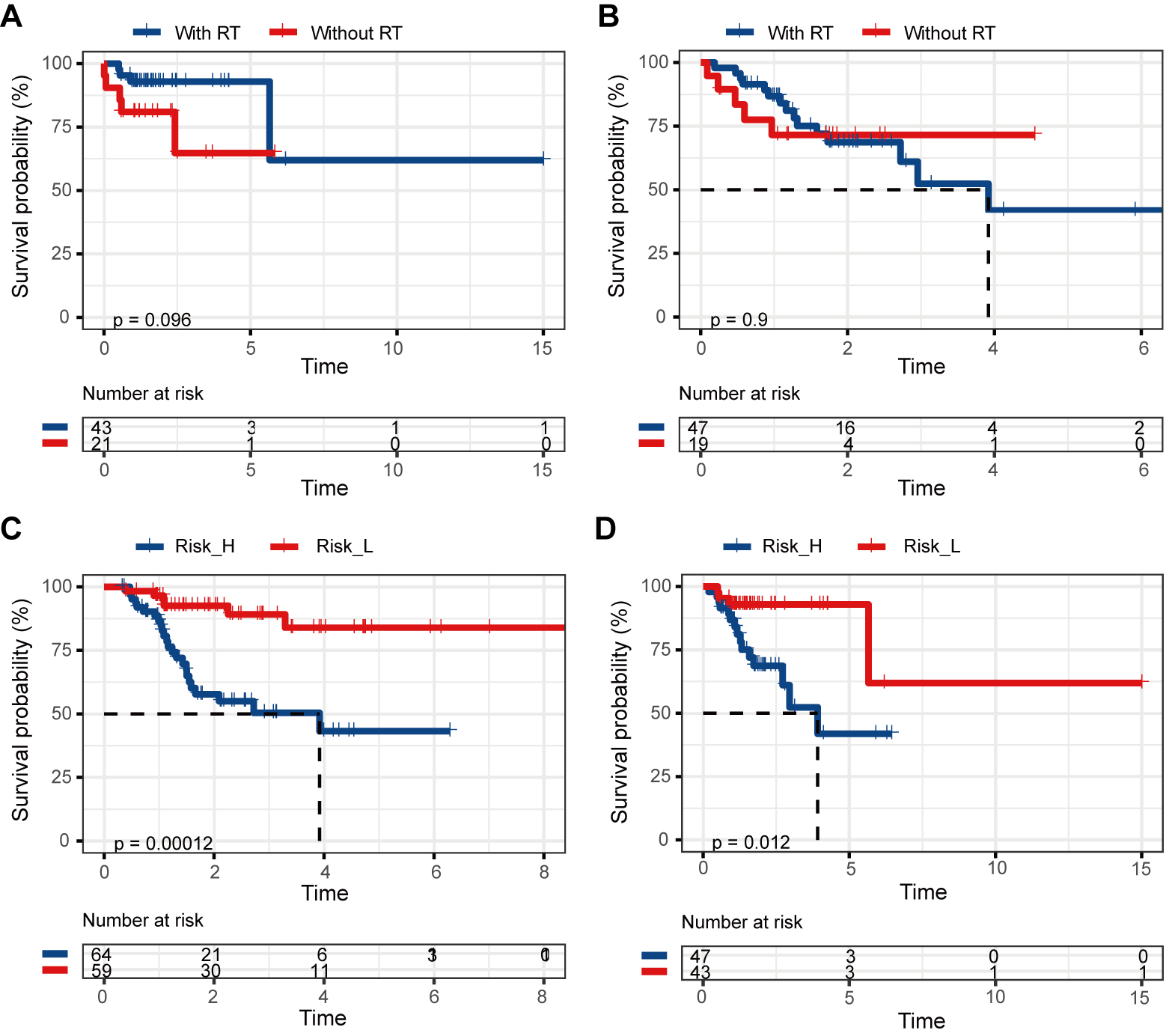


**Supplementary Figure 3.** The survival analysis of HNSCC patients with chemotherapy or radiotherapy (RT). Kaplan-Meier analysis for OS in (A) low- and (B) high-risk groups with the application of RT. And the predictive value of risk model for OS in patients with (C) chemotherapy and (D) radiotherapy.

## Supplementary Tables

| **Table S1.** Introduction of 10 MRGs constructing the prognostic model. | | | |
| --- | --- | --- | --- |
| Gene symbol | Full name | Encoding protein | Risk Coefficient |
| ADA | Adenosine deaminase | An enzyme that catalyzes the hydrolysis of adenosine to inosine | 0.013053726 |
| ALG3 | Alpha-1, 3-mannosyltransferase | A member of the ALG3 family, a crucial class of glycosyltransferases | 0.004785301 |
| CHPF2 | Chondroitin polymerizing factor 2 | A kind of glycosyltransferase used for chondroitin synthesis | 0.005102212 |
| CYP4X1 | Cytochrome P450, family 4, subfamily X, polypeptide 1 | A member of the cytochrome P450 superfamily of enzymes | -0.009745387 |
| GATM | Glycine amidinotransferase | A mitochondrial enzyme that belongs to the amidinotransferase family | -0.011462085 |
| GPT | Glutamic-pyruvate transaminase | Cytosolic alanine aminotransaminase 1 | -0.004815018 |
| HPRT1 | Hypoxanthine phosphoribosyltransferase 1 | A transferase that converts hypoxanthine and guanine into adenosine and guanosine | 0.012284735 |
| HS3ST1 | Heparan sulfate (glucosamine) 3-O-sulfotransferase 1 | A member of the heparan sulfate biosynthetic enzyme family | 0.054555857 |
| HSD17B6 | Hydroxysteroid (17-beta) dehydrogenase 6 | A member of the retinol dehydrogenase family | -0.003982598 |
| LIPE | Lipase, hormone-sensitive | One of the lipolytic enzymeson that regulates adipose  tissue adiposetissue deposition | -0.070739922 |
| The introduction of genes from GEPIA v2.0 database. | | | |
